# Age-dependent variations in rumen bacterial community of Mongolian cattle from weaning to adulthood

**DOI:** 10.1186/s12866-022-02627-6

**Published:** 2022-09-07

**Authors:** Anum Ali Ahmad, Jianbo Zhang, Zeyi Liang, Mei Du, Yayuan Yang, Juanshan Zheng, Ping Yan, RuiJun Long, Bin Tong, Jianlin Han, Xuezhi Ding

**Affiliations:** 1 Key Laboratory of Yak Breeding Engineering, Lanzhou Institute of Husbandry and Pharmaceutical Sciences, Chinese Academy of Agricultural Science, Lanzhou, 730050 China; 2 State Key Laboratory of Grassland Agro-Ecosystems, School of Life Sciences, Lanzhou University, Lanzhou, 730000 China; 3 Key Laboratory of Veterinary Pharmaceutical Development, Ministry of Agricultural and Rural Affairs, Lanzhou Institute of Husbandry and Pharmaceutical Sciences, Chinese Academy of Agricultural Science, Lanzhou, 730050 China; 4 The State Key Laboratory of Reproductive Regulation and Breeding of Grassland Livestock, The Research Center for Laboratory Animal Science, School of Life Sciences, Inner Mongolia University, Mongolia, China; 5 CAAS-ILRI Joint Laboratory on Livestock and Forage Genetic Resources, Institute of Animal Science, Chinese Academy of Agricultural Sciences (CAAS), Beijing, China; 6 Livestock Genetics Program, International Livestock Research Institute (ILRI), Nairobi, Kenya

**Keywords:** Mongolian cattle, Rumen microbiota, Volatile fatty acid, Age-dependent microbiota, Post-weaning

## Abstract

**Background:**

Rumen microbes play an important role in ruminant energy supply and animal performance. Previous studies showed that the rumen microbiome of Mongolian cattle has adapted to degrade the rough forage to provide sufficient energy to tolerate the harsh desert ecological conditions. However, little is known about the succession of rumen microbes in different developmental stages of post-weaning Mongolian cattle.

**Methods:**

Here, we examined the succession of the rumen microbial composition and structure of 15 post-weaning Mongolian cattle at three developmental stages i.e., 5 months (RM05), 18 months (RM18) and, 36 months (RM36) by using the 16S rRNA gene sequencing method.

**Results:**

We did not find any age-dependent variations in the ruminal concentrations of any volatile fatty acid (VFA) of Mongolian cattle. The diversity of the rumen bacterial community was significantly lower in RM05 group, which reached to stability with age. Bacteroidetes and Firmicutes were the two dominant phyla among all age groups. Phylum Actinobacteria was significantly higher in RM05 group, phyla Spirochaetes, and Tenericutes were highly abundant in RM18 group, and phyla Proteobacteria and Epsilonbacteraeota were enriched in RM36 group. Genera *Prevotella_1*, *Bacteroides*, and *Bifidobacterium* were abundant in RM05 group. The short chain fatty acid (SCFA) producing bacteria *Rikenellaceae_RC9_gut_group* showed high abundance in RM18 group and fiber degrading genus *Alloprevotella* was highly abundant in RM36 group. Random forest analysis identified *Alloprevotella*, *Ileibacterium*, and *Helicobacter* as important age discriminatory genera. In particular, the genera *Ruminococcaceae_UCG-005*, *Bacteroides*, *Saccharofermentans*, and *Fibrobacter* in RM05, genera *[Eubacterium] coprostanoligenes_grou*p, *Erysipelotrichaceae_UCG-004*, *Helicobacter*, *Saccharofermentans*, *Papillibacter*, and *Turicibacter* in RM18, and genera *Rikenellaceae_RC9_gut_group*, *Lachnospiraceae_AC2044_group*, and *Papillibacter* in RM36 showed the top interactions values in the intra-group interaction network.

**Conclusions:**

The results showed that rumen microbiota of Mongolian cattle reached to stability and maturity with age after weaning. This study provides some theoretical evidence about the importance of functional specific rumen bacteria in different age groups. Further studies are needed to determine their actual roles and interactions with the host.

## Background

Ruminants have a complex rumen microbial community to help in adaptation to high fiber plants and provide energy in the form of VFA for the growth of the host by fermenting nutrients [1]. Rumen microbial community is known to be influenced by various factors such as diet, age, genetics, breed, and geography [2–4]. These factors directly or indirectly influence rumen microbiota that responds to variations in the environment and might change the physiological response of the host.

The development of rumen microbiota is linked to the structural variations of the rumen with age. The relationship between host and rumen microbiota occurs at birth as vertical transmission of microbes from the mother and is considered a crucial route for the establishment of microbiota in newborns [5]. Prior study on the occurrence of rumen microbial communities in newborn revealed rapid colonization of the rumen by aerobic and facultative anaerobic microbial taxa close to birth, which gradually replaced by exclusively anaerobic taxa between 6 and 8 weeks of age [6]. The appearance of cellulolytic bacteria in 3–5 day-old animals was observed, which then became common in 2–3 week-olds [7]. A study of the ruminal microbial communities of three 14-day-old pre-ruminant calves and three 42-day-old pre-ruminant calves reported the presence of bacteria and functions observed in adult animals [8]. A study conducted on bovine from birth to adulthood (2 years old) reported the influence of age on the rumen bacterial community [9]. Another study on Holstein cattle from 9 to 120 months of age described age-related variations in the rumen microbial community [10]. Similarly, the rumen microbiota of pre-ruminant calves fed milk replacer was also characterized [11]. Weaning calves from liquid to solid has shown rapid changes in their rumen microbiota [12, 13]. However, fewer studies have reported the effect of age on the rumen microbial community of the host animal in the ruminant after weaning [14–16].

The studies on rumen microbial communities of calves reported the use of young animals with a small age gap to study age-related changes in the rumen microbiota [11, 17] without considering possible differences between young and adult animals. So, in this study, we extended the age gap with the aim of determining the age-dependent maturation of the specific microbiota in post-weaning ruminants from young to adulthood.

Mongolian cattle are one of the most distinctive livestock breeds native to Mongolia and Inner Mongolia, while few of them exist in the north, northeast, and northwest regions of China [18]. It is well preserved due to its unique geographical environment and is the only surviving treasure among local beef cattle breeds in China [19]. It has adapted to the semi-arid and low winter temperature of the Mongolian Plateau. It has been herded by nomads for centuries and is highly regarded for its high-quality meat [20]. Mongolian cattle primarily feed on grasses, roughage, and organic feed, and live on plains and grasslands [21]. In comparison to Holstein calves which are separated from their dam soon after their birth, fed milk replacer, and weaned at 7–8 weeks by concentrate and hay, Mongolian calves remain with their dam after birth, are fed mothers’ milk, and naturally weaned at 5^th^ month of age [22, 23]. So, their time of weaning, environmental condition, and food or diet used for weaning are different. Previous studies mainly focused on the health, metabolism, and productivity of Mongolian cattle [19, 24, 25]. The changes in composition and structure of bacterial communities at different developmental stages of Mongolian cattle from weaning to adulthood remain untouched. So, this study aimed to identify age-dependent variations in the structure and composition of rumen bacteria in three different age groups of Mongolian cattle from weaning to adulthood by using the 16S rRNA gene sequencing method and correlated it with rumen fermentation parameters. We hypothesized that the rumen bacterial community and fermentation parameters will change significantly at different developmental stages of Mongolian cattle from weaning to adulthood. This study will help to understand the possible differences in rumen microbiota between young and adult animals and to understand the interactions among microbes in different age groups of cows. It will further help in modulating microbial communities to enhance the productivity of animals.

## Results

### Body weight and rumen volatile fatty acid analysis

We observed significant difference in body weight of RM05 and RM36 group, while RM18 showed no significant difference with RM05 and RM36 groups. We did not find any age-dependent variations in the ruminal concentrations of any of the VFA of Mongolian cattle (Table 1). However, the total VFA present in the rumen of Mongolian cattle increased with age.

**Table 1:** Age-dependent variations in rumen VFA concentrations of Mongolian cattle

Items	RM05	RM18	RM36	SEM	P-Value
Body weight (kg)	92.2^a^	144.7^ab^	195.5^b^	14.02	0.01
Total VFA (mM)	40.92	44.75	47.66	2.74	0.67
Acetate (mM)	28.88	32.22	34.59	1.99	0.61
Propionate (mM)	6.59	7.18	7.27	0.42	0.76
Isobutyrate (mM)	0.97	0.83	0.90	0.05	0.40
Butyrate (mM)	2.77	3.08	3.32	0.26	0.61
Isovalerate (mM)	1.40	1.14	1.26	0.09	0.44
Valerate (mM)	0.29	0.28	0.31	0.03	0.93
Acetate/Propionate	4.34	4.51	4.79	0.10	0.22
Acetate/Butyrate	12.04	11.02	9.63	0.52	0.44

*RM05* 5 months, *RM18* 18 months, *RM36* 36 months, *SEM* Standard error of mean. Values are presented as mean ± SEM

### Sequencing data and bacterial diversity analysis

**Fig. 1:**
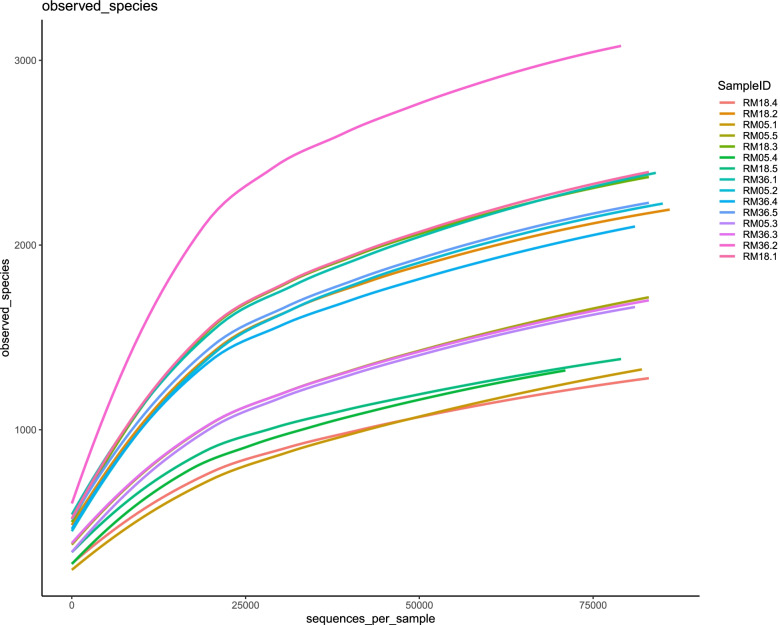
Rarefaction curves of observed species in three different age groups of Mongolian cattle

A total of 1,235,273 high-quality reads were obtained with an average of 82,351 sequence reads per sample from 15 rumen samples of Mongolian cattle after filtering and removal of chimera sequences. The rarefaction curve for observed species reached the plateau, displaying sufficient sequencing depth to correctly describe the composition of each sample (Fig. 1). The Good’s coverage was more than 99.1% with the average length of sequence reads of 416 bp. Overall 9,084 OTUs were detected based on 97% nucleotide sequence identity and 2,346 were shared among three groups. While 1,145, 1,117, and 2,397 specific OTUs were present in RM05, RM18, and RM36, respectively.

**Fig. 2:**
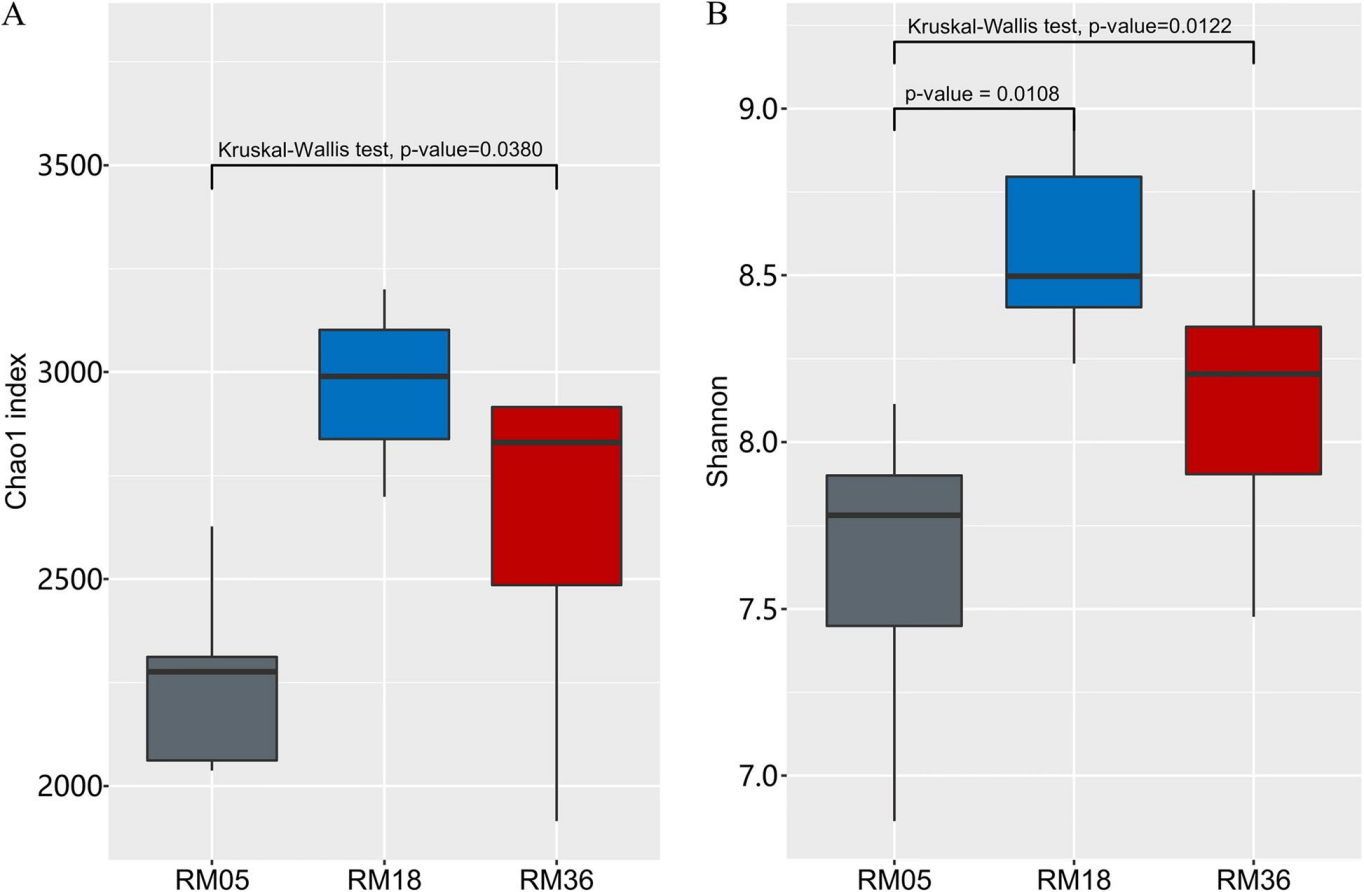
Age-dependent variations in rumen alpha bacterial diversity of Mongolian cattle. **A** Chao index and **B**) Shannon index. RM05, 5 months; RM18, 18 months; RM36, 36 months

Chao1 and Shannon indices showed significant variations (*P* < 0.05) among different age groups (Fig. 2A and B). Chao1 index was significantly lower in the RM05 group compared to RM36 group, while no significant variations were observed between RM05 and RM18 and between RM18 and RM36 groups. Shannon index was significantly lower in RM05 compared to RM18 and RM36 and no significant difference was observed between RM18 and RM36 groups.

**Fig. 3:**
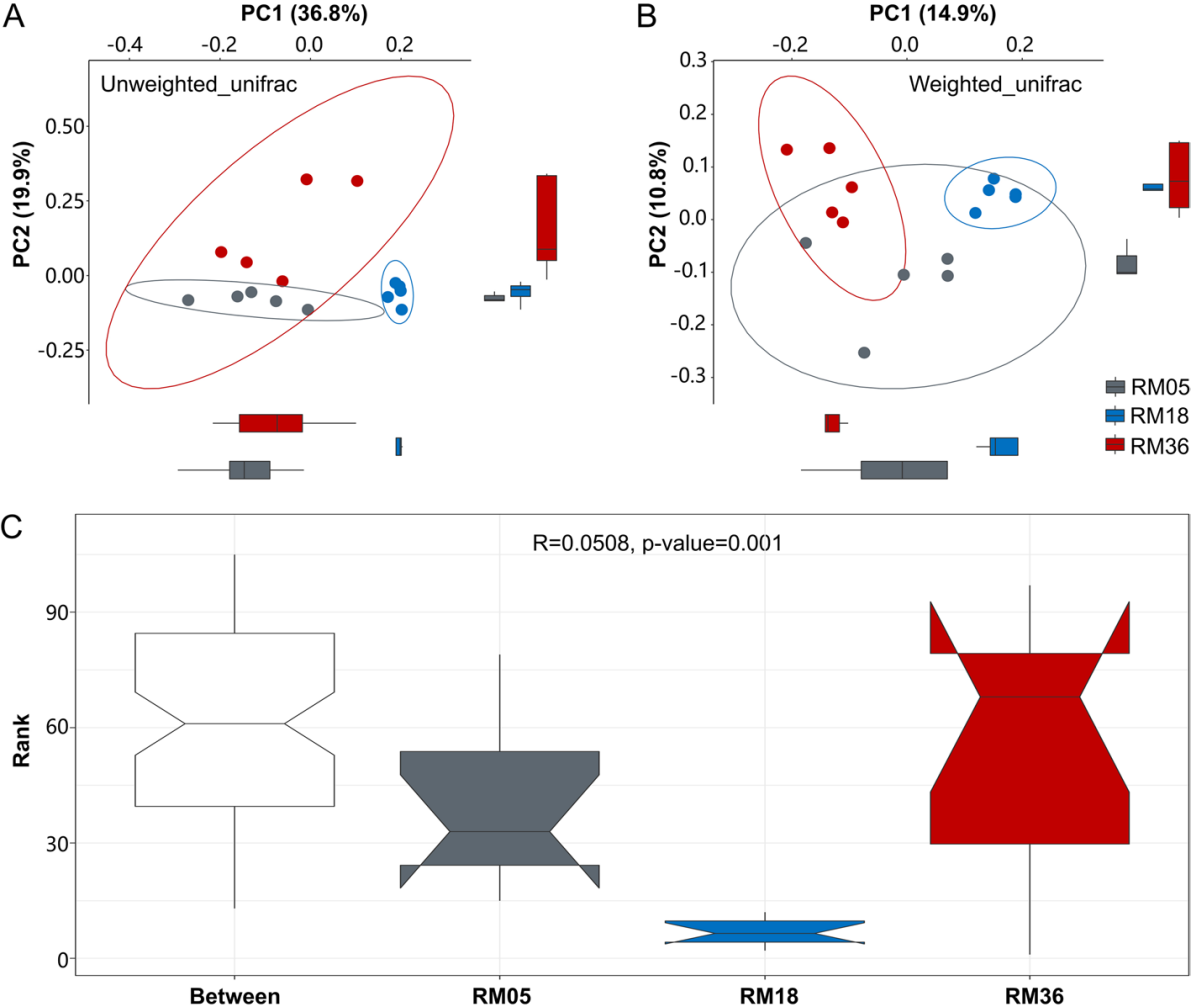
**A** Principal coordinate analysis (PCoA) based on unweighted Unifrac distance, **B**) PCoA based on weighted Unifrac distance, and **C**) Anosim analysis indicating age-dependent variations in rumen bacterial diversity. X-axis, 1^st^ principal component, and Y-axis, 2nd principal component. Different colors represent different groups. RM05, 5 months; RM18, 18 months; RM36, 36 months

To further analyze the variations in the bacterial community among groups, we plotted PCoA graphs based on unweighted and weighted UniFrac distance (Fig. 3A and B). Both PCoA plots showed separate and clear clustering of samples based on age groups. ANOSIM analysis showed significant differences in rumen bacterial composition at OTU level between RM05 and RM18 (*R* = 0.65, *P* = 0.007) groups, between RM18 and RM36 (*R* = 0.524, *P* = 0.007) groups, and between RM36 and RM05 (*R* = 0.356, *P* = 0.007) groups (Fig. 3C).

### Dominant rumen bacterial community

**Fig. 4:**
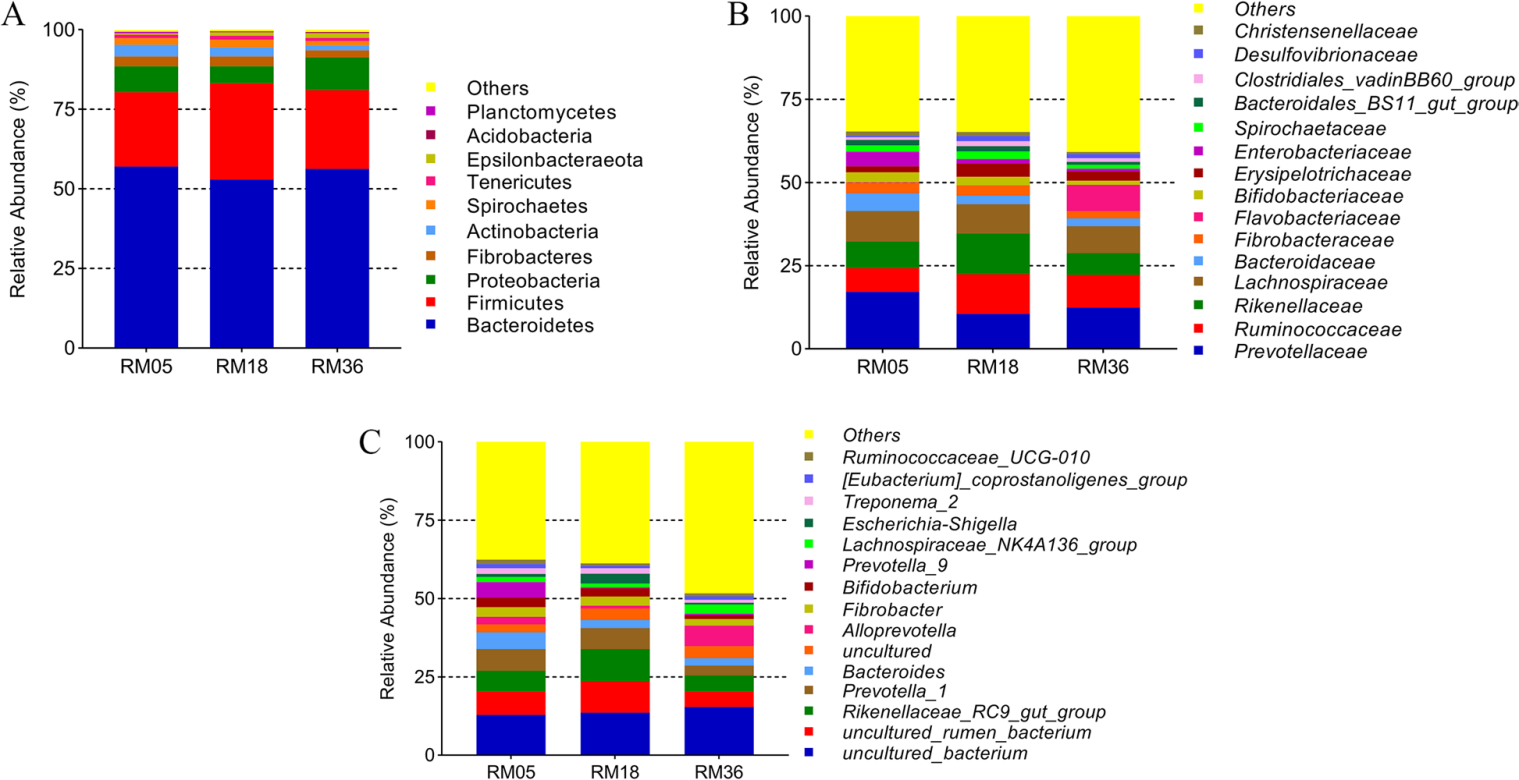
Bargraph showing the core rumen bacterial composition at phylum, family and genus levels present in three different age groups of Mongolian cattle

A total of 28 phyla, 27 families, and 631 genera were identified in rumen samples of Mongolian cattle. The phyla Bacteroidetes (55.49%) and Firmicutes (26.0%) were found to be the dominant phyla in all age groups accounting for 70–73% of total reads (Fig. 4A). The other core phyla included Proteobacteria (8.06%), Fibrobacteres (2.74%), Actinobacteria (2.71%), Spirochaetes (1.91%), Tenericutes (1.09%), Epsilonbacteraeota (0.98%), Acidobacteria (0.23%), and Planctomycetes (0.16%).

At the family level, *Prevotellaceae* (13.51%) and *Ruminococcaceae* (9.56%) were the dominant families among the three age groups (Fig. 4B). *Rikenellaceae* (8.79%), *Lachnospiraceae* (8.73%), *Bacteroidaceae* (3.54%), *Fibrobacteraceae* (2.74%), *Flavobacteriaceae* (2.73%), *Bifidobacteriaceae* (2.32%), *Enterobacteriaceae* (2.22%) and *Spirochaetaceae* (1.80%) were other major families.

*Uncultured_bacterium* (13.9%), *uncultured_rumen_bacterium* (7.33%) and *Rikenellaceae_RC9_gut_group* (7.29%) were dominant genera in three age groups (Fig. 4C). Other major genera included *Prevotella_1* (5.62%), *Bacteroides* (3.54%), *Uncultured* (3.33%), *Alloprevotella* (3.25%), *Fibrobacter* (2.67%), *Bifidobacterium* (2.31%), and *Prevotella_9* (2.31%).

### Age-dependent variations in rumen bacterial community

**Fig. 5:**
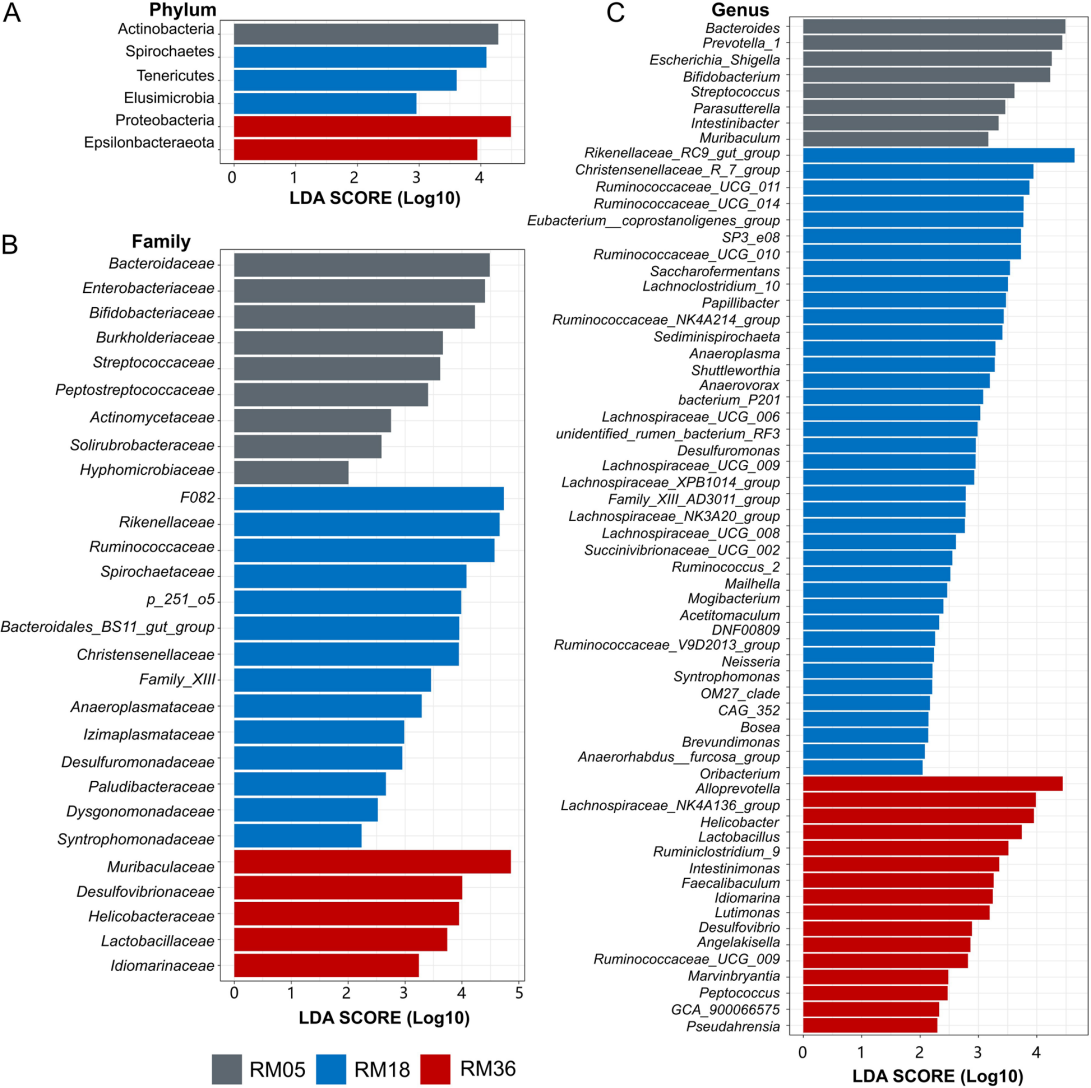
Age-dependent variations in rumen bacterial composition of Mongolian cattle indicated by LEfSe analysis. Variations at **A**) phylum level, **B**) family level, and **C**) genus are shown. RM05, 5 months; RM18, 18 months; RM36, 36 months. The length of the bar column represents the LDA score

We used LeFSe analysis to identify variations at phylum (Fig. 5A), family (Fig. 5B), and genus levels (Fig. 5C) with age. The abundance of phylum Actinobacteria was significantly higher in the RM05 group, while phyla Proteobacteria and Epsilonbacteraeota were enriched in the RM36 group. The phyla Spirochaetes, Elusimicrobia, and Tenericutes were highly abundant in the RM18 group compared to the RM05 and RM36 groups.

At the family level, *Bacteroidaceae*, *Enterobacteriaceae*, *Bifidobacteriaceae*, and Streptococcaceae were abundant in the RM05 group. Families *F082*, *Rikenellaceae*, *Ruminococcaceae*, *Spirochaetaceae*, and *Christensenellaceae* were enriched in the RM18 group, while *Muribaculaceae*, *Desulfovibrionaceae*, *Helicobacteraceae*, and *Lactobacillaceae* were highly abundant in RM36 group. The rest of the significantly varied families are presented in Fig. 5B.

Among the top 10 genera, *Prevotella_1*, *Bacteroides, Escherichia_Shigella*, *Bifidobacterium*, and *Streptococcus* showed significantly higher abundance in RM05 compared to RM18 and RM36 groups. Genera *Rikenellaceae_RC9_gut_group*, *Christensenellaceae_R.7_group*, *Ruminococcaceae_UCG.011*, and *Ruminococcaceae_UCG.014* were enriched in the RM18 group, while genera *Alloprevotella, Lachnospiraceae_NK4A136_group, Helicobacter*, and *Lactobacillus* were significantly higher in RM36 group. Significantly enriched minor genera in three age groups are shown in Fig. 5C.

### Random forest analysis to determine age discriminatory bacterial genera

**Fig. 6:**
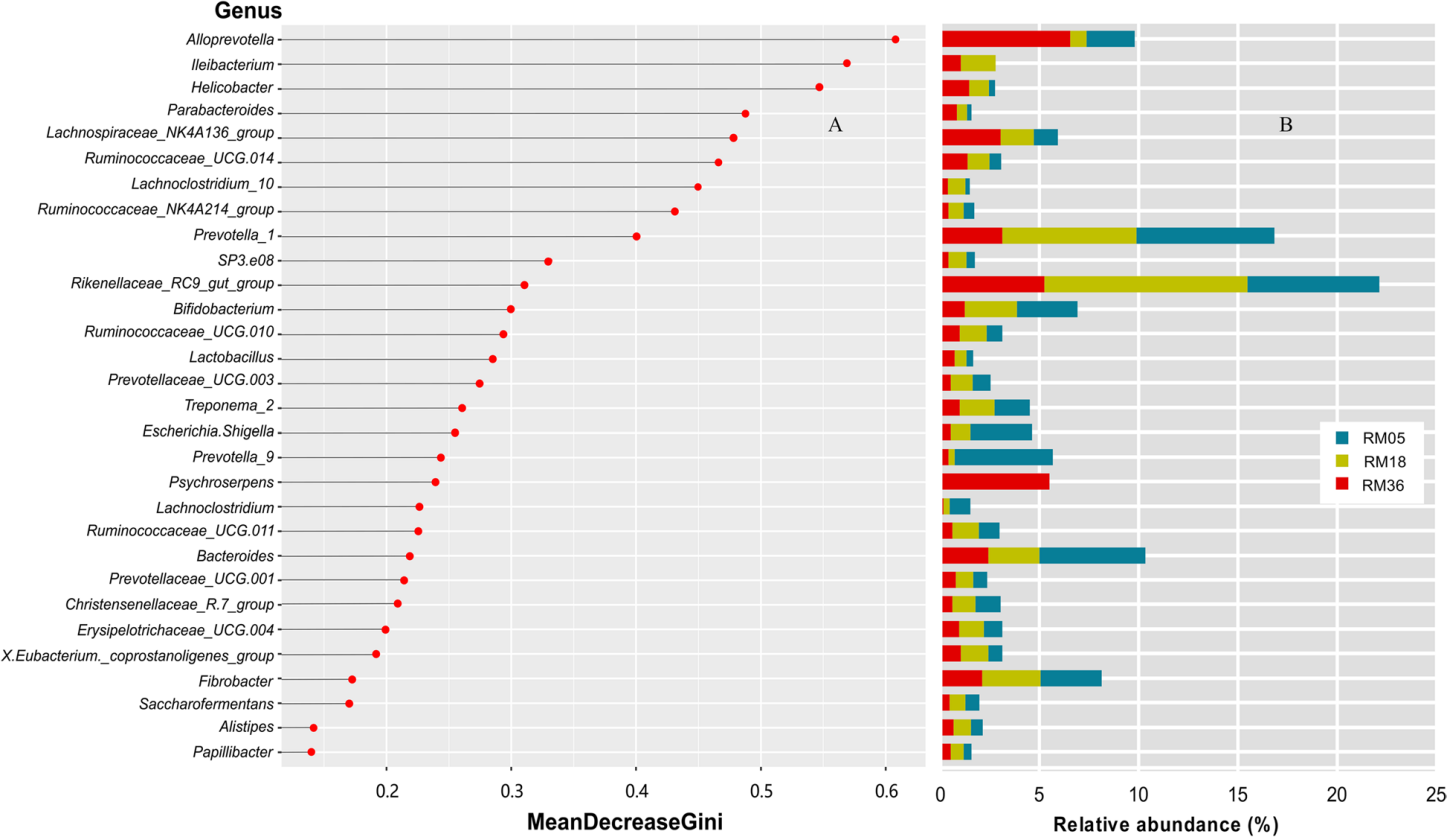
**A** Random forests analysis of rumen bacterial genera using data from three age groups of Mongolian cattle. Genera with large values of mean decrease Gini are considered as the important predictor variable. **B** Bar plots showing the relative abundance of age discriminatory genera according to age group

Random forest analysis classified 30 bacterial genera based on their mean decrease Gini scores were classified as important age discriminatory genera (Fig. 6). Among the top 30 bacterial genera, *Alloprevotella*, *Ileibacterium,* and *Helicobacter* were selected as impactful predictors based on their larger values. Genera *Alloprevotella* and *Helicobacter* showed high abundance in RM36, while genu *Ileibacterium* was highly abundant in the RM18 group. The rest of the discriminatory genera and their abundance in three age groups are shown in Fig. 6. Generally, the selected 30 bacterial genera highlight their potential importance in selected age groups.

### Intra-group interactions of bacterial communities

**Fig. 7:**
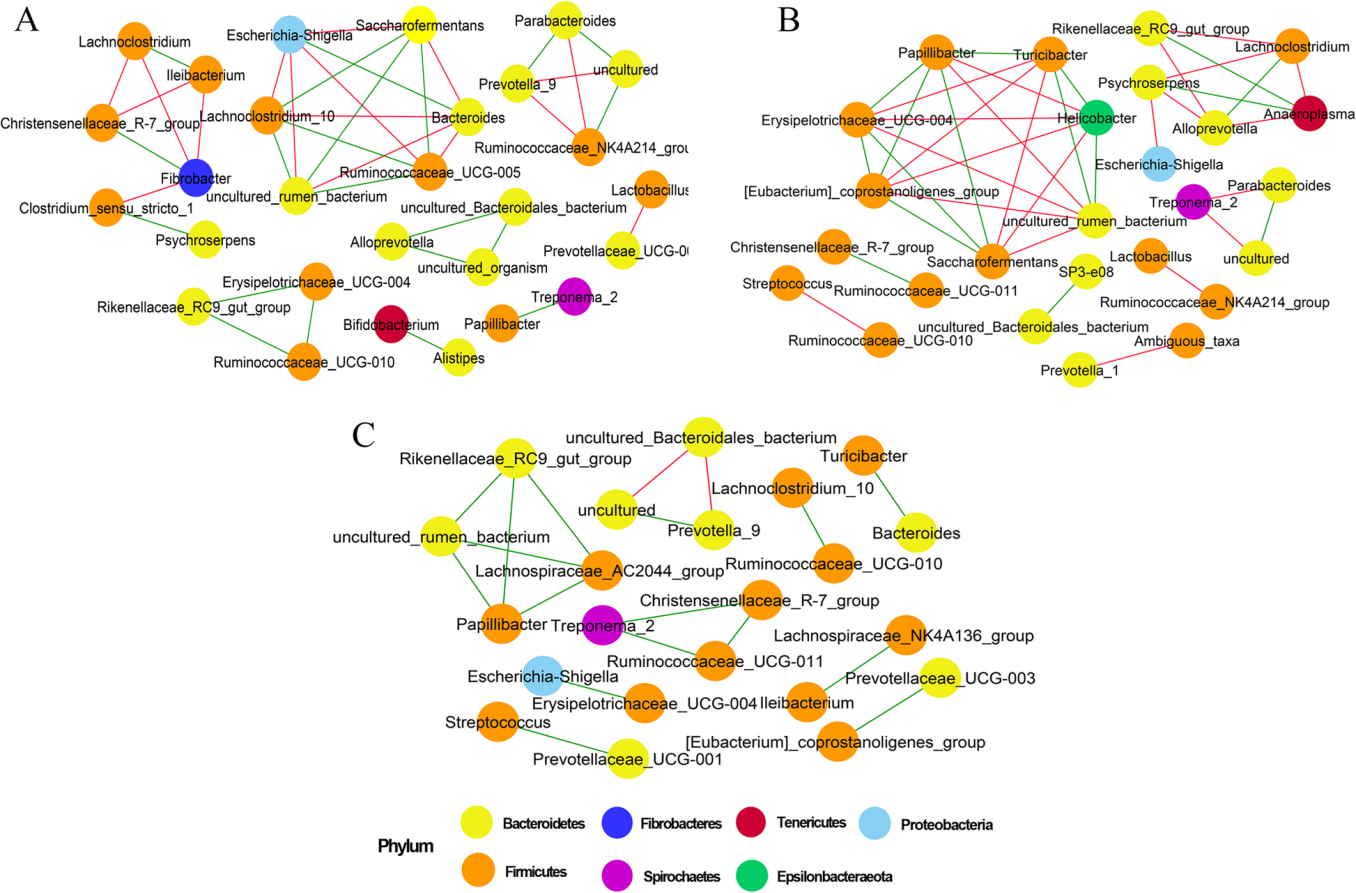
Correlation network of bacterial genera of Mongolian cattle in **A** RM05, **B** RM18, and **C** RM36 groups. Each node represents bacterial genera. Red lines show a negative correlation while green lines indicate a positive correlation between genera. RM05, 5 months; RM18, 18 months; RM36, 36 months

We explored the intra-group interactions of bacterial genera in three age groups based on Spearmen correlation associations (Fig. 7). All genera in the networks were assigned to phyla Bacteroidetes, Firmicutes, Fibrobacteres, Spirochaetes, Tenericutes, Proteobacteria, and Epsionobacteraeota. We observed more number of interactions in the RM18 group as compared to RM05 and RM36, while fewer interactions were observed in RM36.

In RM05, the genera *uncultured_rumen_bacterium* (interactions = 5), *Ruminococcaceae_UCG-005* (interactions = 5), *Bacteroides* (interactions = 5), *Escherichia-Shigella* (interactions = 5), *Saccharofermentans* (interactions = 5), and *Fibrobacter* (interactions = 4) were identified to play critical role with high number of interactions in the network. The genera *Saccharofermentans*, *Lachnoclostridium_10*, and *Ruminococcaceae_UCG-005* (*R* = -0.1) showed positive and negative correlations with *uncultured_rumen_bacterium* and *Bacteroides*, respectively. The genus *Escherichia-Shigella* displayed negative and positive correlations with *uncultured_rumen_bacterium* and *Bacteroides*, respectively. The genera *Saccharofermentans*, and *Lachnoclostridium_10* were positively while *Escherichia-Shigella* was negatively correlated with *Ruminococcaceae_UCG-005*. The genus *Escherichia-Shigella* showed a negative correlation with *Saccharofermentans*, and *Lachnoclostridium_10*, while a positive correlation was observed between *Saccharofermentans* and *Lachnoclostridium_10*.

In RM18, genera *uncultured_rumen_bacterium* (interactions = 6), *[Eubacterium] coprostanoligenes_group* (interactions = 6), *Erysipelotrichaceae_UCG-004* (interactions = 6), *Helicobacter* (interactions = 6), *Saccharofermentans* (interactions = 6), *Papillibacter* (interactions = 6), and *Turicibacter* (interactions 6) displayed high interactions in the network. Genera *Saccharofermentans, [Eubacterium]_coprostanoligenes_group*, *Erysipelotrichaceae_UCG-004* (*R* = 0.81), and *Papillibacter* were negatively correlated, while *Helicobacter* and *Turicibacter* were positively correlated with *uncultured_rumen_bacterium*. Genera *Saccharofermentans, Erysipelotrichaceae_UCG-004* (*R* = 0.81), and *Papillibacter* displayed positive correlation while *Helicobacter* and *Turicibacter* showed negative correlation with *[Eubacterium] coprostanoligenes_group*. Genus *Turicibacter* was negatively correlated with *Erysipelotrichaceae_UCG-004*. While *Saccharofermentans* and *Papillibacter* were positively and negatively correlated with *Erysipelotrichaceae_UCG-004* and *Helicobacter*, respectively. Genus *Turicibacter* showed a positive correlation with *Papillibacter* and *Helicobacter* and a negative correlation with genu *Saccharofermentans*. Genus *Papillibacter* was positively correlated with *Saccharofermentans*.

In RM36, genera *uncultured_rumen_bacterium* (interactions = 3), *Rikenellaceae_RC9_gut_group* (interactions = 3), *Lachnospiraceae_AC2044_group* (interactions = 3), and *Papillibacter* (interactions = 3) showed high interactions in the network. All these genera showed positive relationship with each other.

### Correlation of dominant genera with body weight and VFA

**Fig. 8:**
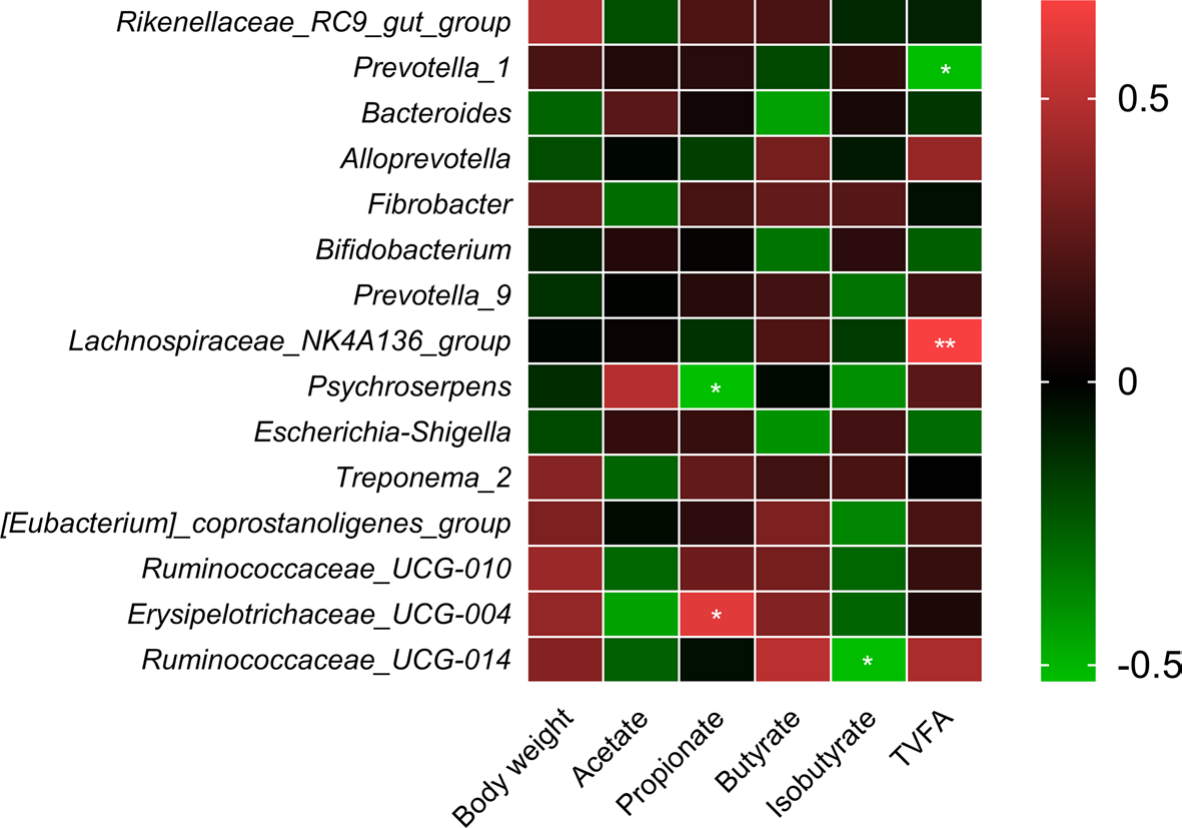
Correlation of top 15 bacteria genera with body weight and rumen VFA. Red color indicates positive correlation and green color shows negative correlation. * =  < 0.05, and ** =  < 0.001. TVFA, total volatile fatty acid

The relations of the top 15 bacterial genera with body weight and VFA were investigated using Spearman correlations coefficients (Fig. 8). The genera *Erysipelotrichaceae_UCG-004* (*R* = 0.61, *P* = 0.01) and *Psychroserpens* (*R* = -0.53, *P* = 0.04) were positively and negatively correlated with propionate, respectively. Genus *Ruminococcaceae_UCG-014* (*R* = -0.52, *P* = 0.04) showed negative correlation with isobutyrate. The genera *Prevotella_1* (*R* = -0.52, *P* = 0.04) and *Lachnospiraceae_NK4A136_group* (*R* = 0.67, *P* = 0.007) were negatively and positively associated with TVFA, respectively.

## Discussion

Age has been known to be a major factor influencing rumen microbiota in animals [26]. It is therefore important to determine the effects of age on rumen fermentation and the microbial community to improve our understanding of age-related changes. In this study, we characterized the rumen bacterial community and fermentation parameters in Mongolian cattle at three different developmental stages from weaning to adulthood. Just like yak, Mongolian cattle have adapted to high altitude, rough forage, and extreme environmental conditions and might have developed unique rumen microbiota to assist in the adaptation. The rumen microbiota of Mongolian cattle underwent substantial changes by the weaning strategy at 5 months of age, and core microbiota appears to reach to maturity and stability with age. We also identified functional specific bacteria in three age groups.

In our study, the proportions of VFA increased with increasing age, however these changes were not significant among three age-dependent, which is consistent with a study conducted on sheep and cattle [10, 27]. The rapid absorption of VFA through rumen epithelium into the bloodstream might be related to insignificant variations of rumen VFA in Mongolian cattle. Moreover, as all the animals fed the same diet, we suggested that the variations in the bacterial composition might be due to age-related physiological changes.

The composition of rumen microbiota is known to differ at different developmental stages. The rumen microbiota of yak from birth to 12 years of age exhibited age-related variations and maturation [28]. Studies conducted on cattle and goats reported significant changes in the rumen microbial diversity with age [10, 26], which is consistent with our results. We recorded high bacterial diversity in the RM18 group as compared to the other two groups. We speculated that increased diversity of the rumen microbiota might be associated with the enrichment of bacterial genera responsible for the metabolism of complex polysaccharides in high fiber pasture grass to help in the adaptation and survival of animals that became stable and mature with age.

We found a core microbiota consisting of Bacteroidetes, and Firmicutes as the two dominant phyla in the studied groups, which is consistent with the previous reports on cattle [29]. A study reported that rumen microbiota undergoes stability and maturity after the first 6 weeks of birth [30]. However, we found significant variations in the core microbiota of studied age groups. Similar findings were also reported in animals from the 6-month and 2-year groups receiving the same diet, indicating that the rumen microbiota undergoes developmental changes that are independent of diet [17]. Other studies also displayed that the bacterial community composition continued to evolve and mature with age [10, 31, 32]. In this study, a high abundance of phylum Actinobacteria in the rumen at a young age (RM05) might be helpful in consuming a variety of vegetation available in the hard desert environment due to its ability to decompose all sorts of organic matters such as cellulose, lignin, and chitin [33]. A high abundance of phyla Spirochetes, Tenericutes, and Elusimicrobia were present in the RM18 group. Phylum Spirochetes is known to ferment plant polymers such as pectin, xylan, and arabinogalactan [34], while phylum Tenericutes is capable of degrading lignin [35]. Phyla Elusimicrobia plays important role in the fermentation of sugars and nitrogen metabolism required for the efficient productivity of animals (Méheust et al. 2020). These phyla might be involved in the adaption of the Mongolian cattle to a variety of substrates for the generation of energy. In the RM36 group, we found a high abundance of Proteobacteria and Epsilonbacteraeota. Phylum Proteobacteria plays important role in maintaining anaerobic environment of rumen by decreasing redox potential and in turn aids in colonization of strict anaerobes [36]. While, phyla Epsilonbacteraeota is famous for its role in reducing nitrite and nitrate to generate energy [37]. These phyla might be associated in enhancing productivity of Mongolian cattle with maturity by providing energy.

We found functional-specific bacterial genera at different development stages of Mongolian cattle. We identified significantly high abundance of *Prevotella_*1, *Bacteroides*, and *Bifidobacterium* in RM05 group. *Prevotella* is known to be a beneficial genus due to its relationship with a plant-rich diet suggesting [38]. *Bacteroides* generate energy by fermenting a wide variety of sugar derivatives from the plant material and also provide resistance to infections [39]. While, Lactic acid-producing genus *Bifidobacterium* is known as beneficial bacterial and provides protection against enteric infection due to synergistic adhesion effect [40, 41]. This genus can utilize starch, amylopectin, maltotriose, and maltodextrin to produce lactic acid [42]. These genera might be playing important role in the growth, health, and development of Mongolian cattle at a young age. The genus *Rikenellaceae_RC9_gut_group*, highly abundant in RM18 group, is known to produce propionate, succinate, butyrate, and acetate which serve as an important energy source for the ruminal epithelial cells and helps in regulating rumen function [43]. The genus *Alloprevotella* was highly abundant in RM36 group, which is famous for its ability to produce acetic acid and succinic acid as the end product of fermentation of plant fiber [44]. *Alloprevotella* was also highlighted as the potential biomarker by random forest.

Intra-group bacterial interactions are vital to the structure and dynamics of the rumen bacterial community [261]. Firmicutes was the most abundant phylum in all the networks, indicating that this phylum is suited to a wide range of environmental conditions. The fact that *uncultured_rumen_bacterium*, *Ruminococcaceae_UCG-005*, *Bacteroides*, *Escherichia-Shigella*, *Saccharofermentans*, and *Fibrobacter* displayed top interactions in RM05 indicates their importance in this age group. *Ruminococcaceae_UCG.010* and *Fibrobacter* are known to facilitate the degradation of cellulose and hemicellulose in the rumen of animals [45, 46], while *Saccharofermentans* plays important role in the fermentation of a variety of several carbohydrates and produce fumarate, lactate, and acetate [47]. *Escherichia-Shigella* is a potential pathogen known to delay the establishment of the anaerobic rumen environment [48]. The high interactions of this genus might be the result of the suppressed immune system due to the stress of weaning in young animals. In the RM18 group, *[Eubacterium] coprostanoligenes_group*, *Erysipelotrichaceae_UCG-004*, *Helicobacter*, *Saccharofermentans*, *Papillibacter*, and *Turicibacter* displayed top interactions. Genus *Eubacterium coprostanoligenes* is known to convert cholesterol into coprostanol and influence the fat metabolism of the host [49, 50]. *Erysipelotrichaceae_UCG-004*, *Saccharofermentans*, and *Papillibacter* produce acid-enhancing metabolites that lower the rumen pH and cause the death of gram-negative pathogenic bacteria such as *Helicobacter* as indicated by the negative correlation in the network [51–54]. We speculated that top interacting genera might be involved in coping with pathogenic bacteria introduced due to the suppressed immunity during the weaning in animals and also providing energy to help animals to adapt and survive the high fiber content present in pasture grass. We observed less interaction in the RM36 group and only *Rikenellaceae_RC9_gut_group*, *Lachnospiraceae_AC2044_group*, and *Papillibacter* were top interacting genera. *Rikenellaceae_RC9_gut_group* is known to increase when roughage content increases in diet [55]. The other two genera are known to produce butyrate by fermenting complex polysaccharides fiber and influence the rumen development and health of animal [56]. Overall, we assumed that these genera might be aiding in digestion of high fiber diet and maintaining the health of Mongolian cattle. The networks provide new dimensions to our understanding of the age-dependent variations in rumen bacterial community interactions by identifying keystone taxa.

Although three different age groups received the same diet, the variations in bacterial abundance might indicate that at 5 months of age rumen microbiota undergoes developmental changes independent of diet. The presence of Short-chain fatty acids producing bacteria in RM18 might be linked to its role in providing energy to the growing animals and helping them to survive on shrubs and herbs during the cold and arid environment. While fiber degrading bacteria in RM36 might indicate the establishment of a stable and mature microbial community. The diet of Mongolian cattle is dominated by a variety of shrubs and halophytes, so rumen microbiota must be adapted to degrade such a recalcitrant diet which is rich in lignocellulosic materials. It is noteworthy that the age gap in this study is quite large and perhaps the analysis of smaller age would reveal more detailed variations in rumen microbiota with development.

## Conclusions

In conclusion, the rumen microbiota of Mongolian cattle reached to stability and maturity with age after weaning. The diversity of the rumen bacterial community was lower at a young age which becomes stable with age. Bacteroidetes and Firmicutes were the core phyla in all age groups. We identified functional-specific bacterial genera in three age groups. Genera *Prevotella_*1, *Bacteroides*, and *Bifidobacterium* were abundant in RM05. The Short-chain fatty acids producing bacteria *Rikenellaceae_RC9_gut_group* showed high abundance in the RM18 group and the fiber degrading genus *Alloprevotella* was highly abundant in the RM36 group. The genera *Ruminococcaceae_UCG-005*, *Bacteroides*, *Saccharofermentans*, and *Fibrobacter* in RM05, genera *[Eubacterium] coprostanoligenes_group*, *Erysipelotrichaceae_UCG-004*, *Helicobacter*, *Saccharofermentans*, *Papillibacter*, and *Turicibacter* in RM18, and genera *Rikenellaceae_RC9_gut_group*, *Lachnospiraceae_AC2044_group*, and *Papillibacter* in RM36 showed the top interactions values in the intra-group interaction network. This study provides some preliminary information about the structure and composition of rumen microbiota in Mongolian cattle from weaning to adulthood. Further studies are needed to determine their actual roles and interactions with the host.

## Methods

### Site description

The trial was conducted at Alashan, which is located in the westernmost part of Inner Mongolia Autonomous Region, bordered in the north by Mongolia, in the south and west by Gansu province, China during the winter of 2020. Alashan has a continental climate, which is dry and windy. Winter is cold and summer is hot. Vegetation in this area is dominated by *Salix cupularis*, *Haloxylon ammodendron*, *Caragana jubata* and *Kobresia spp*. The precipitation on the Alashan is under 150 mm/year, while annual temperature ranges from 6 °C-8°C on average.

### Animals and sample collection

Fifteen Ujumqin Mongolian female cattle from three different age groups i.e., 5 months old (RM05), 18 months old (RM18), and 36 months old (RM36) were randomly selected from a farm in Alashan Mongolia and were individually penned until sampling. The selected animals shared the same raising protocols i.e., all animals were naturally weaned at 5 months of age and before weaning they were purely on mother milk. After weaning, animals were allowed to graze the natural alpine shrub grasslands year round and drank water from the local river. None of the studied animals were pregnant or given birth before. The animals used in this study were not genetically related or receiving antibiotic treatment. All the animals were purely grazing and were not provided with any supplements. The body weight of animals from each age group was measured at the time of sample collection by using electronic weighing scale (Shanghai Yaohua Urban Systems Co., Ltd. Shanghai, China).

Animals from RM18 and RM36 were restrained in a veterinary crush before sampling to ensure the safety of animals, while animals from RM05 group were straddled between the handler’s legs and their shoulders were firmly squeezed between legs to avoid movement and misplacement of the oral tube. Rumen content (70 mL/animal, liquid part) was collected using an oral stomach tube from each animal before morning grazing and snap-frozen in liquid nitrogen and then stored at − 80 °C until use. Polyvinyl chloride oral tube (length = 125 cm) with small side holes (7 mm in diameter) located at the insertion end was used for young animals [57]. The length of the tube to be inserted was measured as the distance from the tip of the calf’s nose to the point of its elbow behind the front leg and marked on the tube with a piece of tape i.e., approximately 45 cm. For adult animals, stainless steel rumen fluid extractor (Chengdu Huazhi Kaiwu Technology Co., Ltd., Chengdu, China) was used for sampling and approximately 200 cm of the tube was inserted to reach the center of the rumen. Each time before taking the new sample, the tube was thoroughly cleaned with fresh warm water and about 10-15 ml of the sample from each cattle was always discarded to prevent saliva contamination [58].

### Analysis of rumen volatile fatty acids

The frozen rumen fluid sample was thawed at 4 °C and thoroughly mixed by vortexing. After that, 10 mL of rumen fluid was centrifuged at 3000 g for 10 min, and 1 mL of the supernatant was transferred to a 1.5 mL centrifuge tube, along with 0.2 mL of a metaphosphoric acid solution containing the internal standard 2-ethylbutyric acid. The sample was mixed, placed in an ice-water bath for 30 min, and centrifuged at 10,000 × g at 4 °C. The supernatant was transferred to a new 1.5 mL centrifuge tube and placed at 4 °C for testing. The volatile fatty acids (VFA) concentration was determined by gas chromatography (Agilent Technologies 7820A GC system, Santa Clara, CA) equipped with a 30 m × 0.25 mm × 0.33 μm fused silica column (AE-FFAP, Atech Technologies Co. Ltd., Shanghai, China). The gas chromatographic conditions and subsequent test procedures were conducted as described previously [59].

### DNA extraction and sequencing

Genomic deoxyribonucleic acid (DNA) from rumen fluid was extracted by cetyltrimethylammonium bromide method [60] and pure DNA was eluted in 150 µL of elution buffer and stored at − 20 °C until use. DNA quality and quantity were checked by 1.5% agarose gel electrophoresis and NanoPhotometer® spectrophotometer (Implen, Westlake Village, CA, USA), respectively [61]. Polymerase chain reaction (PCR) amplification of the V3-V4 region of the 16S rRNA gene was performed for bacterial analysis by using universal primer pairs (343F (5'-TACGGRAGGCAGCAG-3')-798R (5'-AGGGTATCTAATCCT-3')) with barcodes [62]. PCR amplification was performed by using Phusion® High-Fidelity PCR Master Mix with GC Buffer from New England BioLabs. Briefly, PCR amplifications were done in duplicate with 25 μL reaction mix containing 2X phusion master mix, 0.5 µM forward and reverse primers, and 20 ng of genomic DNA. The thermal cycling procedure consisted of an initial denaturation step at 98 °C for 30 s, followed by 25 cycles of 98 °C for 10 s, 56 °C for 30 s, and 72 °C for 20 s, and a final extension at 72 °C for 10 min. The amplicons were visualized using 1.5% agarose gel electrophoresis and purified with AMPure XP beads (Agencourt) according to the manufacturer's instructions [63]. The purified products were used for second round of PCR for the enrichment of ampilcons having adapters on both sides using TruSeq™ DNA sample preparation kit (Illumina Inc, San Diego, CA) according to the manufacturer’s protocol and quantified using Qubit dsDNA Assay kit (Thermo Fisher) [64]. Paired-end sequencing was carried out according to the standard protocol using the Illumina HiSeq2500 PE250 method by commercial company (Oebiotech, Shanghai, China) [65].

### Bioinformatics analysis

After sequencing, barcodes and primer sequences were truncated. The QIIME (Quantitative Insights Into Microbial Ecology, Version 1.9.1) software was used to remove low-quality sequences from the raw data to get clean tags [66]. Chimera sequences were removed from clean tags using UCHIME (version 2.4.2) software to get valid tags [67]. Valid tags were clustered into Operational Taxonomic Unit (OTUs) using Vsearch (version 2.4.2) software according to 97% similarity [68]. The representative sequences of the OTUs were used to classify bacterial taxa using against Silva database (Version 123) (https://www.arb-silva.de/) using RDP Naive Bayesian classifier algorithm [69, 70]. Rarefaction curve was constructed in QIIME software, while bargraphs at phylum, family, and genus levels were created using GraphPad Prism version 8.00 for Windows (www.graphpad.com/). The alpha diversity indices such as Chao1, Shannon, Simpson, and Goods-coverage were calculated using QIIME software (Version 1.9.1). Unweighted and weighted uniFrac distance based principal coordinates analysis (PCoA) plots were drawn in R studio (Version 2.15.3) (http://www.rstudio.com/) using vegan package to demonstrate the difference between samples [71]. A correlation heat map was generated in GraphPad Prism version 8.00 for Windows (www.graphpad.com/).

### Statistical analysis

Before any statistical analyses were conducted, all data were checked for normality using Shapiro–Wilk test using SPSS software (Version 20.0, IBM, Armonk, NY, United States). The Kruskal Wallis test was used to compare VFA across different groups using R software (Version 2.15.3) [72]. Analysis of similarities (ANOSIM) analysis was performed by using the ANOSIM function of the R vegan package to confirm statistically significant differences between groups [73]. The linear discriminant analysis effect size (LEfSe) method was used to examine age-dependent variations at phylum and genus levels using a linear discriminant analysis (LDA) score equal to 4 as a thresholds value [74]. Microbial interactions within studied age groups (Intra-group interaction) were determined by Spearman’s correlation coefficient of 40 top rumen bacterial genera to identify keystone species. Only genera showing *P* < 0.05 were further selected to plot the network by using the cytoscape (Version 3.6.13) [75]. Random forest analysis was performed to identify important age discriminatory bacterial genera by using randomForest function in the R package [76]. All P-values were adjusted using false discovery rate to remove false-positive results and significance was declared at *P* < 0.05.

## Data Availability

The datasets supporting the conclusions of this article are available in the National Center for Biotechnology Information (NCBI) repository under BioProject ID PRJNA762300 (https://www.ncbi.nlm.nih.gov/bioproject/762300).
